# The Archaeal Cell Cycle

**DOI:** 10.1146/annurev-cellbio-111822-120242

**Published:** 2024-09-21

**Authors:** Alice Cezanne, Sherman Foo, Yin-Wei Kuo, Buzz Baum

**Affiliations:** Medical Research Council Laboratory of Molecular Biology, Cambridge, United Kingdom

**Keywords:** archaea, cell division, cell cycle, eukaryogenesis

## Abstract

Since first identified as a separate domain of life in the 1970s, it has become clear that archaea differ profoundly from both eukaryotes and bacteria. In this review, we look across the archaeal domain and discuss the diverse mechanisms by which archaea control cell cycle progression, DNA replication, and cell division. While the molecular and cellular processes archaea use to govern these critical cell biological processes often differ markedly from those described in bacteria and eukaryotes, there are also striking similarities that highlight both unique and common principles of cell cycle control across the different domains of life. Since much of the eukaryotic cell cycle machinery has its origins in archaea, exploration of the mechanisms of archaeal cell division also promises to illuminate the evolution of the eukaryotic cell cycle.

## Introduction

The cell cycle regulates the path by which one cell becomes two. This is one of the most fundamental of all biological processes. During each round of the cell cycle, cells must complete several critical processes: They must double their mass, replicate their genome, and physically partition the genome and all other cellular components into the two new cells generated at division. Furthermore, these events must be carefully orchestrated in time and space. While these processes have long been studied in bacteria and eukaryotes, our understanding of the mechanisms orchestrating cell cycle progression in archaea is only just emerging.

One of the main reasons we know so little about the archaeal cell cycle is that archaea were only identified as a third domain of life in the 1970s by Carl Woese and George E. Fox—300 years after van Leeuwenhoek described bacterial and eukaryotic cells (Woese & Fox 1977; see also Eme et al. 2017, Lake 1988, Woese et al. 1990). Since then, interest in the cell biology of archaea has steadily grown. While archaea appear superficially similar to bacteria (e.g., in having a single circular chromosome), some of the molecular machines involved in key steps in the archaeal cell cycle have close counterparts in eukaryotes (Iwabe et al. 1989, Lake et al. 1984). Yet, in other ways, archaea appear fundamentally different from all other forms of life on our planet. Archaeal cells are bounded by a distinct lipid membrane composed of lipids formed from isoprenoid chains linked by ether bonds to a glycerol phosphate head group with the opposite chirality of that found in bacteria and eukaryotes (Albers & Meyer 2011, Boucher et al. 2004, De Rosa & Gambacorta 1988, Koga & Morii 2007). Furthermore, some archaea have a unique chromosome architecture (Badel & Bell 2024). Nevertheless, the information processing machinery most archaea use to replicate, transcribe, and translate information encoded in their genomes is strikingly similar to the equivalent machinery found in eukaryotic cells (Takemata et al. 2019, Zillig et al. 1989).

**Figure 1:**
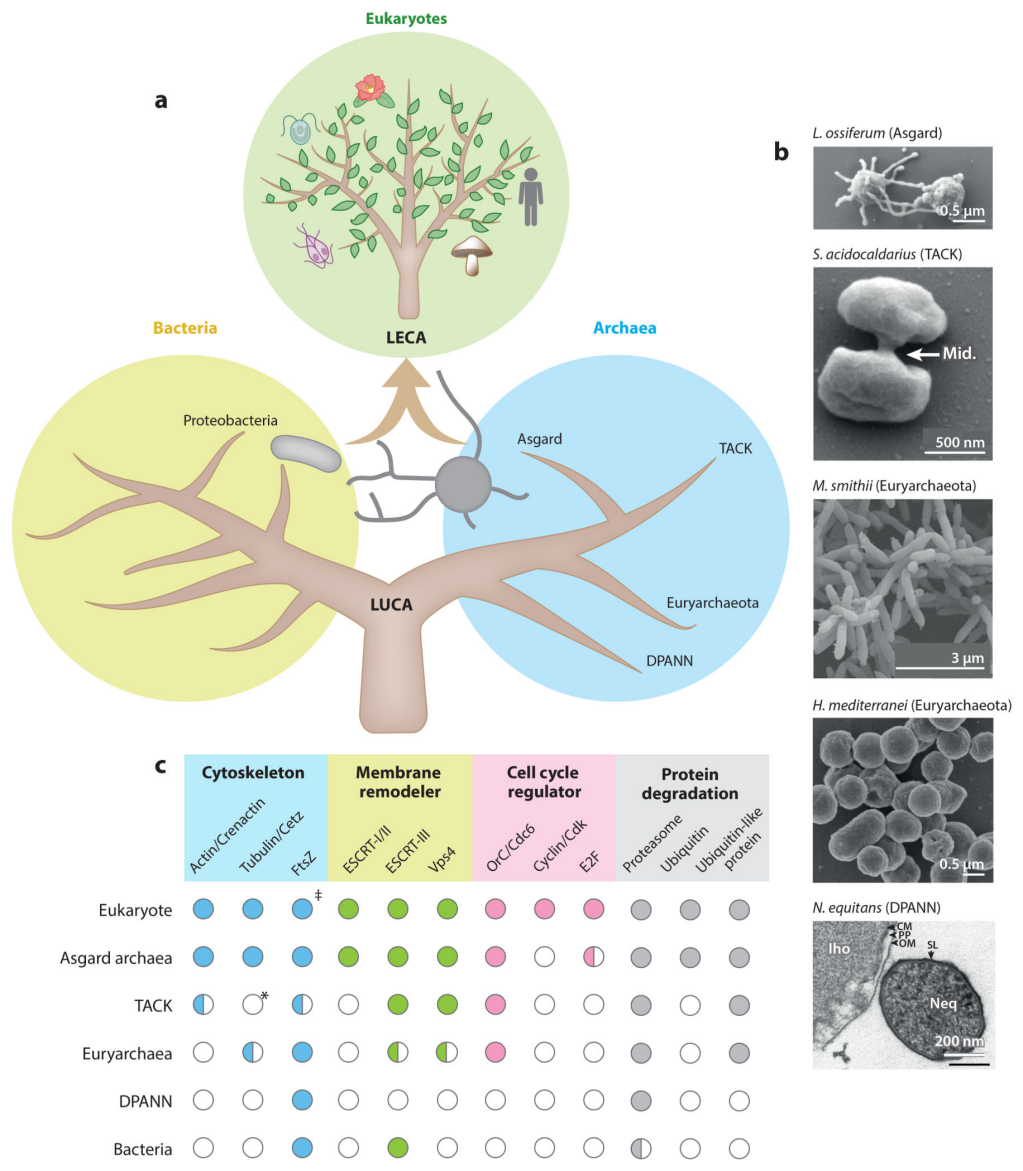
The evolutional diversity of archaea. (*a*) A schematic tree of life showing the major phyla of archaea. The symbiotic relationship between an Asgard archaeon and an alphaproteobacterium has been suggested to have given rise to LECA. (*b*) Representative species from each phylum: *Lokiarchaeum ossiferum, Sulfolobus acidocaldarius, Methanobrevibacter smithii, Haloferax mediterranei*, and *Nanoarchaeum equitans*. (*c*) The distribution of key cell division machinery and cell cycle regulators, showing homologs present in most of the species in the corresponding group (*filled circles*), homologs present in specific subbranches (*half-filled circles*), and homologs absent in the majority of the group (*empty circles*). An asterisk indicates tubulin homologs that were detected in *Candidatus Nitrosoarchaeum limnia* and *Candidatus Nitrosopumilus koreensis* (Thaumarchaeota) (Yutin & Koonin 2012). The double dagger indicates that eukaryotic FtsZs are found mostly in mitochondria and chloroplasts (Osteryoung & Nunnari 2003). Abbreviations: CM, cytoplasmic membrane; Iho, *Ignicoccus hospitalis*; LECA, last common ancestor of eukaryotes; LUCA, last universal common ancestor; mid., midbody; Neq, *N. equitans*; OM, outer membrane; PP, periplasm; SL, S-layer. Images in panel *b* reproduced with permission from Rodrigues-Oliveira et al. (2023) (*L. ossiferum*), Charles-Orszag et al. (2021) (*S. acidocaldarius*), Ruaud et al. (2020) (*M. smithii*), Chen et al. (2019) (*H. mediterranei*) (CC BY 4.0), and Burghardt et al. (2009) (*I. hospitalis* and *N. equitans*).

The first suggestion that archaea are more closely related to eukaryotes than they are to bacteria came from phylogenetic analyses exploring the origins of the core information processing machinery (ribosomal RNA) across the tree of life (Lake 1988, Lake et al. 1984). These data gave rise to the hypothesis that eukaryotes arose from a merger between an alphaproteobacterial cell, which gave rise to the mitochondria, and an archaeal cell that gave rise to the rest of the cell, including the nucleus and cytoplasm (Baum & Baum 2014, Baum & Spang 2023, Eme et al. 2017). This hypothesis is supported by the recent discovery of the Asgard superphylum, which, if one considers core information processing machinery, includes the closest prokaryotic relatives of eukaryotes (Liu et al. 2021, Spang et al. 2015, Zaremba-Niedzwiedzka et al. 2017) (Figure 1*a*,*b*). Strikingly, Asgard genomes also possess a large number of homologs of genes previously thought to be unique to eukaryotes, including genes involved in cell organization (e.g., cytoskeletal filaments) and cell cycle regulators (e.g., the transcription factor E2F) (Eme et al. 2017, 2023; Liu et al. 2021; Williams et al. 2020) (Figure 1c). Since new microbes like Asgard archaea are continuingly coming to light as the result of environmental sampling, we should expect our view of the evolutionary processes that led to the last common ancestor of eukaryotes, the nature of the last universal common ancestor (LUCA), and the evolution of life on Earth to keep changing for some time to come.

## Archaea: A Diverse Domain Of Life

Archaea were initially found in extreme environments such as volcanic hot springs (Barns et al. 1994), deep-sea hydrothermal vents (Takai & Nakamura 2011), arctic environments (Royo-Llonch et al. 2021), and salt lakes (Bhattarai et al. 2021). Since then, advances in molecular detection methods have allowed the additional discovery of archaea across a wide variety of less extreme environments, including soil (Starke et al. 2021), oceans (Könneke et al. 2005), coastal marine environments (DeLong 1992), and sediments (MacLeod et al. 2019), where they play key roles in the nitrogen and carbon cycles (Baker et al. 2020). Archaea have also been detected in the gut microbiome, where their relevance to human health is just beginning to be explored (Geesink & Ettema 2022, Thomas et al. 2022, Youngblut et al. 2021).

Despite being found across diverse environments, archaea remain understudied in part because of the challenges involved in their isolation and cultivation. This is now changing with the development of archaea as model organisms, notably Sulfolobales and Haloarchaea, which serve as representatives of TACK and Euryarchaeota, respectively. This process is aided by developments in imaging techniques (Cezanne et al. 2023, Charles-Orszag et al. 2021, Horn et al. 1999, Pulschen et al. 2020), molecular biology and genetic methods (Wagner et al. 2012, Zink et al. 2020), and cultivation procedures (Cui & Dyall-Smith 2021, Imachi et al. 2020, Klein et al. 2022, Rodrigues-Oliveira et al. 2023), which have expanded the archaeal cell biology toolbox (van Wolferen et al. 2022). In addition, there is a growing body of literature looking at the biology of other diverse archaea that are harder to study, such as DPANN, methanogenic, and Asgard archaea.

Many of the studies looking at archaeal cell biology in recent years have uncovered unique features of archaeal cell growth and DNA replication and division, as well as ways in which these processes resemble those operating in bacteria and eukaryotes. In this review, we aim to bring together these data to give an overview of archaeal cell division.

## The Archaeal Cell Cycle In Context

**Figure 2:**
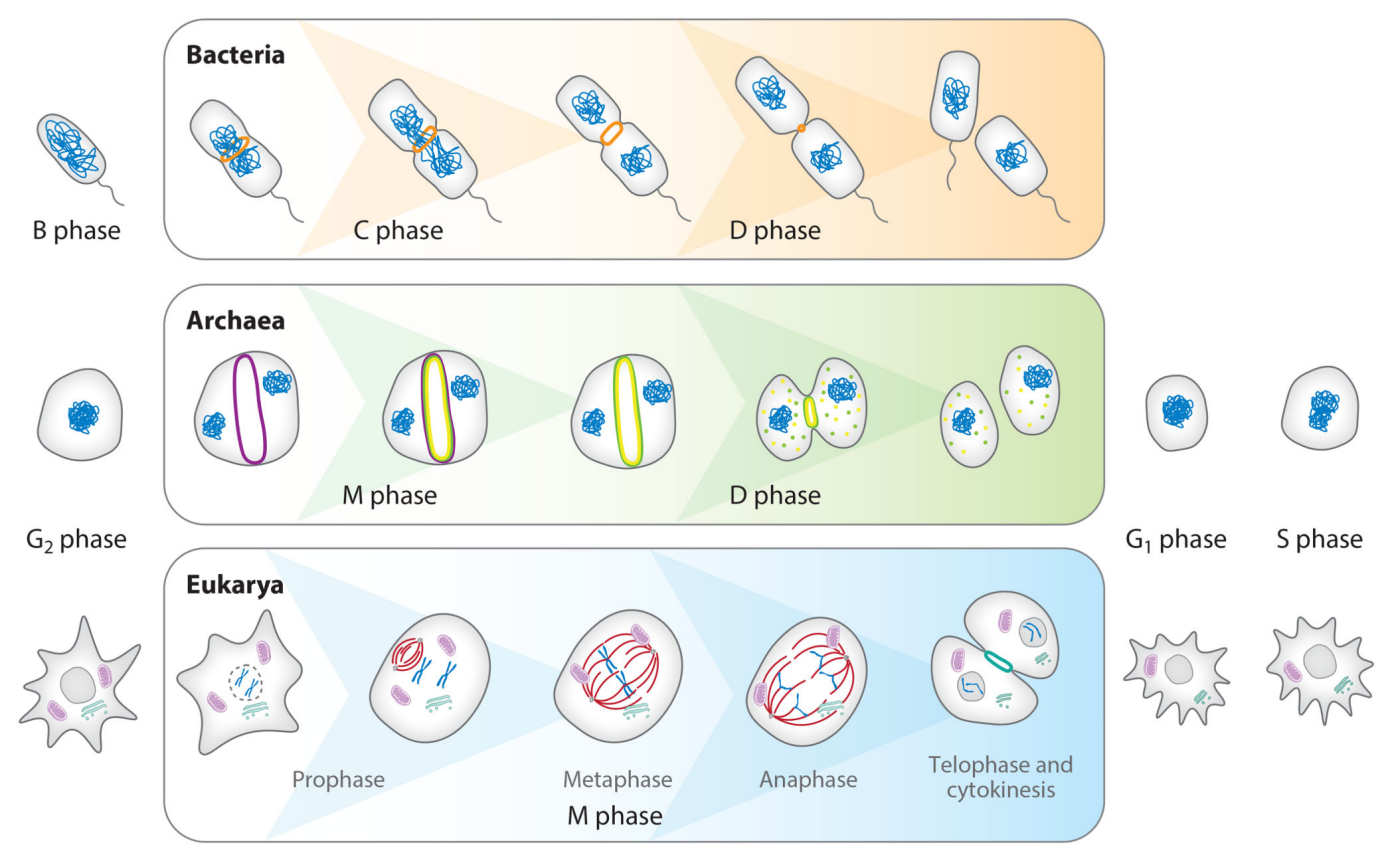
Overview of the cell cycle in representative archaeal, bacterial, and eukaryotic cells. In bacteria, cell division is mediated by the FtsZ ring (*orange*) in the D phase of its life cycle following DNA replication and segregation in the C phase. TACK archaea such as *Sulfolobus acidocaldarius* have defined cell cycle phases similar to those of eukaryotes. Stepwise assembly and disassembly of ESCRT-III polymers CdvB (*purple*), CdvB1 (*green*), and CdvB2 (*yellow*) through proteasome- and Vps4-mediated mechanisms remodel the *Sulfolobus* membrane during cell division (Tarrason Risa et al. 2020). Cyclin-dependent kinases regulate the eukaryotic cell cycle. In animal cells, the degradation of cyclin B triggers anaphase and the separation of sister chromatids (*dark blue*) by microtubules (*red*). The division plane is then assembled across the central part of the spindle, and cytokinesis is induced via the contraction of the actomyosin ring (*turquoise*) to generate two daughter cells.

The cell cycle is best understood in eukaryotes, where it can be thought of in simple terms as the ordered sequence of events by which cells replicate and segregate their DNA. The timing of key events in the cycle is determined by the activity of cyclin-dependent kinases (CDKs) and by checkpoints that cause some later events in the cycle to wait until the completion of early events (Nurse et al. 1998). Eukaryotic cells usually commit to a round of division in the period preceding the onset of DNA replication (S phase). Once DNA synthesis is complete and other critical conditions have been met, cells enter mitosis (M phase). During mitosis, the two copies of the genome are partitioned into two daughter cell nuclei, which are often (but not always) accompanied by division of the rest of the cell. In growing cells, S and M phases tend to be separated by gap phases, termed G_1_ and G_2_ phases, when cells add mass and replicate their mitochondrial genomes (Figure 2).

While these processes are broadly conserved across eukaryotes, they are very different from those described in bacteria. The bacterial cell cycle, which has been studied in most detail in *Escherichia coli* and *Bacillus subtilis*, has historically been divided into three phases: B, C, and D. The B phase spans from the birth of the cell to the onset of DNA replication, the C phase corresponds to DNA replication, and the D phase lies between the completion of DNA replication and cell division (Wang & Levin 2009) (Figure 2). While these can be thought of as corresponding to similar events in eukaryotes, progression through the bacterial cell cycle is regulated very differently. Critically, the progression to each phase in bacteria is not dependent on the completion of the previous phase, as it is in most eukaryotic cells. As a result, cell cycle phases can overlap. For example, in fast-growing *E. coli*, new rounds of DNA replication can be initiated before previous rounds of DNA replication and cell division have been completed (Reyes-Lamothe & Sherratt 2019).

Interestingly, while much remains to be understood about the archaeal cell cycle, phylogenetic analysis along with molecular cell biology studies in members of TACK and Euryarchaeota has revealed that some elements of their cell cycle machinery appear to have bacterial counterparts, whereas others appear more similar to those in eukaryotes.

### The Cell Cycle of TACK and Euryarchaeota

Pioneering work based on model organisms from the order Sulfolobales (part of the TACK archaea superphylum) demonstrated that these archaea have an ordered cell cycle with distinct phases analogous to those observed in eukaryotes (Bernander & Poplawski 1997) (Figure 2). Interestingly, Sulfolobales spend most of their cell cycle in G_2_, with two interlinked copies of the genome (Popławski & Bernander 1997, Robinson et al. 2007). Similarly, other TACK archaea have a short G_1_ and a long G_2_ (Lundgren et al. 2008), which have been suggested to aid DNA repair by homologous recombination in the event of DNA damage (Rhind & Russell 1998). In contrast, *Nitrosopumilus maritimus*, a member of the Thaumarchaeota, appears to have G_1_ and G_2_ phases of similar length (Pelve et al. 2011).

While such an ordered cell cycle requires regulation, it is unclear how this is controlled. TACK archaea do not possess obvious homologs of the machinery governing eukaryotic cell cycle progression or key eukaryotic cell cycle checkpoint proteins [e.g., cyclin, CDK, and the anaphase-promoting complex (APC/C) and its regulators] (Figure 1c). Nevertheless, given the well-ordered cell cycle observed in TACK archaea, it is tempting to speculate that these cells may possess checkpoint-like mechanisms similar to those found in eukaryotes that prevent advancement into the next cell cycle phase until certain conditions have been satisfied. In support of TACK archaea possessing controls that mediate passage from one cell cycle phase to the next, *Sulfolobus* cells can be arrested in the G_2_ phase of the cycle by various stimuli, including growth to stationary phase (Bernander & Poplawski 1997), acetic acid or antibiotic treatment (Hjort & Bernander 2001, Lundgren et al. 2004), conditional genetic mutants (Bernander et al. 2000), and inhibition of the proteasome (Tarrason Risa et al. 2020). Additionally, UV-irradiation-induced DNA damage leads to downregulation of cell division genes (Fröls et al. 2007, Lindås et al. 2008, Schult et al. 2018). Furthermore, proteasome inhibition in *Sulfolobus acidocaldarius* inhibits both cell division and DNA replication licensing (Tarrason Risa et al. 2020), implying that a common machinery coordinates entry into cell division as well as initiation of the following S phase.

In contrast to TACK archaea, Euryarchaeota tend to be polyploid (Breuert et al. 2006, Hildenbrand et al. 2011, Malandrin et al. 1999, Marie et al. 1996, Spaans et al. 2015). DNA copy number varies across species and is affected by growth conditions such as the availability of nutrient sources (Breuert et al. 2006, Malandrin et al. 1999). In Haloarchaea, multiple genome copies are replicated throughout the cell cycle (Zerulla & Soppa 2014), suggesting that these cells may not have a distinct S phase. Similarly, the methanogen *Methanococcus jannaschii* appears to have relaxed cell cycle control, with cells displaying a broad range of DNA copy numbers (Malandrin et al. 1999). In contrast, *Methanothermobacter thermautotrophicus* cycles between diploid and tetraploid states, with a very brief G_2_ phase separating DNA replication from chromosome segregation (Majerník et al. 2005). Thus, the mechanisms controlling cell cycle organization vary widely across Euryarchaeota.

Although this discussion highlights differences between members of TACK and Euryarchaeota, they also possess shared mechanisms of cell cycle control. For example, the inhibition of DNA synthesis, through either drug treatment or conditional mutants, causes cell cycle arrest in *Halobacterium salinarum* as it does in TACK archaea (Herrmann & Soppa 2002, Hjort & Bernander 2001). Interestingly, haloarchaeal cells containing multiple copies of the chromosome also fail to divide when treated with the DNA replication inhibitor aphidicolin (Forterre et al. 1984, Herrmann & Soppa 2002, Schinzel & Burger 1984). This finding implies that multiple chromosome copies do not suffice for division and that a new round of DNA replication may be a prerequisite for cell division regardless of how many copies are already present. The converse, however, does not apply. *S. acidocaldarius* and *Haloferax volcanii* cells that are unable to complete division can still undergo further rounds of DNA replication (Darnell et al. 2020; Hurtig et al. 2023; Liao et al. 2021a,b), suggesting that the DNA replication cycle may not depend on successful cell division. Interestingly, the same is true of eukaryotic cells, where the inhibition of cytokinesis does not block entry into the following S phase. Instead, the coupling of M and S phases is achieved via the degradation of mitotic cyclins, which triggers DNA segregation (and nuclear division) and origin relicensing in preparation for the next round of DNA replication (Basu et al. 2022, López-Avilés et al. 2009), resetting the cell cycle clock.

### Cell Cycle Control in Archaea

Despite these early insights into the organization of the archaeal cell cycle, the molecular mechanisms driving cell cycle progression in archaea are still not well understood. Since many TACK archaea possess an ordered cell cycle but lack CDK-cyclin homologs, it is possible that the evolution of cell cycle control preceded the evolution of the eukaryotic cell cycle machinery. While it is unclear what such an ancient cell cycle control system might look like, cyclic transcription (Baumann et al. 2007, Lundgren & Bernander 2007) and proteolysis (Tarrason Risa et al. 2020) appear to play central roles in the regulation of the archaeal cell cycle, analogous to their roles in eukaryotes. In synchronized cultures of *S. acidocaldarius* (Lundgren & Bernander 2007) and *H. salinarum* (Baumann et al. 2007), multiple transcripts exhibit periodic waves of expression. These include transcripts for cell division proteins, such as ESCRT-III homologs (Wittenberg & Reed 2005), as well as DNA replication machinery, such as Cdc6 and DNA polymerase that peak at division in *S. acidocaldarius* and accumulate prior to G_1_/S in eukaryotes (Baum et al. 1998).

While it is far from clear how cyclic transcription is regulated, the archaeal transcription machinery appears to be a mosaic of eukaryotic and bacterial features. These include a basal transcription apparatus similar to that operating in eukaryotes: an RNA polymerase that resembles a stripped-down version of the eukaryotic RNA polymerase II (Huet et al. 1983, Werner & Grohmann 2011) and initiation factors, TATA-box binding protein (TBP) and transcription factor B (TFB), similar to those used in eukaryotes (Langer et al. 1995, Werner 2007). In addition, both TACK archaea and Euryarchaeota possess a large number of bacterial-like transcription factors that are responsible for regulating the expression of specific target genes (Bell & Jackson 1998, Blombach et al. 2019, Koonin et al. 1997, Wenck & Santangelo 2020). These include a set of transcription factors that have been implicated in the regulation of cyclic genes. In Haloarchaea, a ribbon-helix-helix (RHH) domain–containing protein, CdrS, acts as a key transcription regulator for several cell division genes, including the tubulin homolog FtsZ, and its perturbation causes cell division defects (Darnell et al. 2020, Liao et al. 2021b). In addition, the overexpression of an RHH family transcription factor in the TACK archaeon *Saccharolobus islandicus*, aCcr1, inhibits the expression of CdvA (a template protein for the ESCRT-III-based division ring), thereby preventing successful division (Li et al. 2023, Yang et al. 2023). Since genes whose expression is controlled by the RHH family transcription factors constitute only a small portion of cell cycle–specific genes identified to date, a full understanding of the mechanisms giving rise to the waves of gene expression that occur across the archaeal cell cycle remains to be discovered. It is interesting to note here that TACK archaea also possess TFB2, a transcription factor with a cyclin fold that undergoes cyclic expression as cells progress through the cell cycle (Lundgren & Bernander 2007, Noble et al. 1997). In addition, some Asgard archaeal genomes have been reported to have homologs of the E2F transcription factor (Spang et al. 2015, Zaremba-Niedzwiedzka et al. 2017) (Figure 1c), which is the key driver of the transition from G_1_ to S phase in eukaryotes and is regulated by Rb, a protein that also contains a cyclin fold (Chemes et al. 2013). This finding hints at the possible deep conservation of cell cycle–dependent transcription between TACK/Asgard archaea and eukaryotes.

### DNA Replication

Like bacteria, most archaea contain circular chromosomes. While that might imply that archaea regulate DNA replication and segregation in a similar manner to bacteria, this is not the case. The majority of archaeal species studied appear to replicate their genome using multiple replication origins. Sulfolobales, for example, tend to use three origins (Lundgren et al. 2004). Although all three of these origins fire nearly simultaneously during a normal cell cycle, none is essential individually (Samson et al. 2013). The same appears to be the case for *Pyrobaculum calidifontis*, which harbors four origins of replication (Pelve et al. 2012). By contrast, archaea with high ploidy, such as *Haloferax* and *Thermococcus* species, thrive in the absence of all replication origins under laboratory conditions (Hawkins et al. 2013, Norais et al. 2007). In their absence, cells replicate their genomes via a recombination-based mechanism involving RadA, which is conserved across all three domains of life: RecA in bacteria and Rad51 in eukaryotes (Gehring et al. 2017, Hawkins et al. 2013). This raises the question of whether a recombination-based DNA replication mechanism could predate the split that separates the acquisition of bacterial and archaeal machinery involved in origin firing.

Strikingly, the machinery regulating origin firing in most archaea appears more similar to the machinery that unwinds DNA and initiates DNA replication in eukaryotes than to the equivalent protein machine, DnaA, in bacteria (Arias-Palomo et al. 2013). This is the case even for archaea with a single origin of replication, such as *Pyrococcus abyssi*, a member of Euryarchaeota (Matsunaga et al. 2001, 2007; Myllykallio et al. 2000). Origin firing in archaea tends to be triggered by a homolog of the eukaryotic origin binding protein Cdc6/Orc1, which is encoded by a gene located next to the origin upon which it acts (Edgell & Doolittle 1997, Feng et al. 2021, Liu et al. 2000). Cdc6/Orc1 is thought to form a homohexameric complex (making it simpler than its heterohexameric counterpart in eukaryotes) that binds at a single well-defined AT-rich origin of replication prior to its firing. Cdc6/Orc1 then undergoes an irreversible reaction to open the DNA and initiate DNA replication. While Cdc6/Orc1 loading is regulated by cyclic transcription in both archaea and eukaryotes, eukaryotes employ CDK-cyclin-dependent phosphorylation of Cdc6/Orc1 and its partner proteins to ensure that origin firing happens only once per cycle. It is not yet clear how this process is regulated in archaea, although Cdc6/Orc1 may undergo an autophosphorylation event that renders origin firing irreversible (Grabowski & Kelman 2001). While it is not understood how *Sulfolobus* cells limit DNA replication to one round per cycle, the cyclic expression patterns of kinases, *cdc6* genes, and TFB2 hint at the existence of regulatory machinery in archaea that functions to achieve the same ends as CDK-cyclin-dependent regulation in eukaryotes (Lundgren & Bernander 2007).

Archaeal Cdc6/Orc1 is likely aided by a homolog of Cdt1 in *Saccharolobus solfataricus* (Robinson & Bell 2007). This may help recruit the minichromosome maintenance (MCM) helicases, which form two replication forks, moving in opposite directions to open the DNA in front of the replication fork (Kelman et al. 2020). In addition, the archaeal prereplication complex also employs homologs of Cdc45 and GINS (Makarova et al. 2012). Together, this Cdc45-MCM-GINS complex unwinds AT-rich DNA at the origin, leading to the formation of single-stranded DNA that is then bound by single-stranded DNA binding proteins to prevent it from reannealing to form double-stranded DNA (Kelman & Kelman 2014). DNA replication in archaea is also carried out by a DNA polymerase similar to that found in eukaryotes (Edgell & Doolittle 1997). DNA primase, polymerase, and the rest of the replication machinery are recruited to the open origin, enabling the bidirectional elongation of two strands of DNA. The leading strand is extended continuously while the lagging strand is synthesized discontinuously as Okazaki fragments due to the antiparallel nature of DNA. We point interested readers to a number of excellent in-depth reviews of DNA replication in archaea (Greci & Bell 2020, Kelman & Kelman 2014, Kelman & White 2005).

### DNA Segregation

In contrast to eukaryotes, which segregate their multiple linear chromosomes using a tubulin-based mitotic spindle, most archaea appear to possess genes similar to the bacterial chromosome segregation machinery that is used to segregate circular chromosomes and plasmids. In *Sulfolobus*, DNA segregation appears to be a relatively rapid process that occurs shortly before cell division (Pulschen et al. 2020). While it is not known how this is achieved, a ParA-like Walker-type ATPase, SegA, is thought to be recruited to the nucleoid via its interaction with the DNA-binding protein SegB. This SegA/SegB complex has been proposed to compact the DNA in preparation for division (Kalliomaa-Sanford et al. 2012, Yen et al. 2021). However, it is not yet understood how these proteins drive DNA segregation nor whether they are essential for this process. Although SegA and SegB are subject to cyclic transcription, it is not known how DNA segregation is tied to cell division and other events in the cell cycle.

Like other archaea, Asgard archaea have single circular chromosomes and possess homologs of ParA and ParB, which they may use for DNA segregation (Zaremba-Niedzwiedzka et al. 2017). While Asgard genomes encode homologs of eukaryotic tubulin as well as other proteins that fall within the FtsZ protein family, there are no indications that they have anything like a microtubule-based spindle. Thus, for the moment, the evolutionary origins of the microtubule-based spindle appear unclear.

## Cell Division In Archaea

The final steps in the cell cycle are cell constriction and abscission—the process that executes the final cut to separate the two daughter cells. To ensure that both daughter cells inherit a single copy of the genome, the timing and spatial organization of DNA segregation and the recruitment of envelope remodeling machinery and division must be tightly coupled.

**Figure 3:**
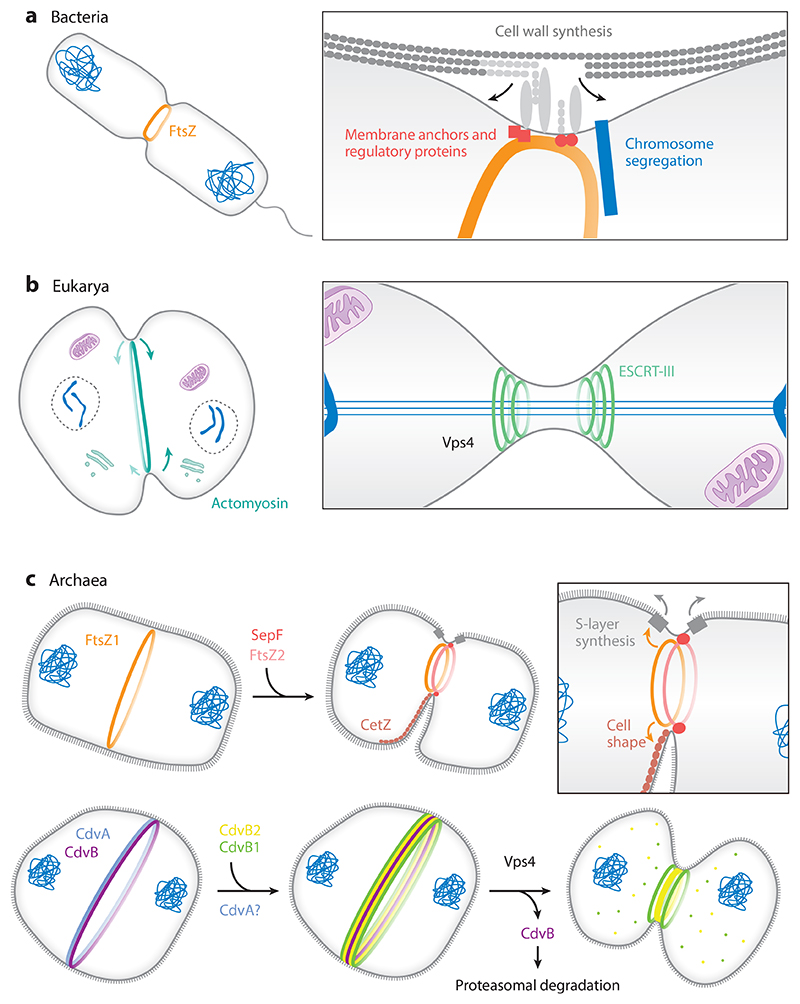
Summary of the different means of cell division in representative bacteria, eukaryotes, and archaea. (*a*) In bacteria, local remodeling and de novo synthesis of the peptidoglycan cell wall together with membrane deformation by the FtsZ ring drive cell division. (*b*) Eukaryotes utilize an actomyosin contractile ring to power cleavage furrow formation, followed by local membrane remodeling by the ESCRT-III machinery to complete cytokinesis. (*c*) (*Top*) In archaea such as *Haloferax volcanii*, FtsZ-mediated division utilizes two homologs of FtsZ together with a membrane anchor, SepF. Localization of the cell shape–regulating machinery CetZ and other membrane envelope and S-layer synthesis machinery suggest a link between cell shape determination and division in these organisms. (*Bottom*) In *Sulfolobus acidocaldarius*, cell division is accomplished by the stepwise assembly and disassembly of the ESCRT-III homologs CdvB, CdvB1, and CdvB2, together with an archaeal-specific CdvA protein that forms a template ring at the site of division. Abbreviation: S-layer, surface layer.

In most bacteria, division is executed by the tubulin homolog FtsZ, which acts together with its membrane anchor SepF to recruit the multiprotein divisome complex. The divisome coordinates DNA segregation with cell wall synthesis, which provides the membrane constricting force for division (Cameron & Margolin 2024) (Figure 3). In the most widely studied eukaryotes, on the other hand, a contractile actomyosin ring is used to guide cell wall deposition (fungi) and membrane constriction (protists and animals). In the best-studied cases, the actomyosin division ring is assembled at mitotic exit and is positioned at the center of the anaphase spindle by a cue emanating from overlapping antiparallel spindle microtubules (Pollard & O’Shaughnessy 2019) (Figure 3).

Interestingly, in both bacteria and eukaryotes, the contraction of the division ring is not sufficient to induce abscission. In bacteria, the FtsZ ring is disassembled prior to complete closure (Söderström et al. 2014), and it is not yet known how the process is driven to completion. Similarly, in animal cells the constriction of the actomyosin division ring ends with the generation of a relatively large midbody, which acts as a physical barrier between the two nascent daughter cells. The final step in the process of division is then mediated by a family of conserved ESCRT-III polymers (Liu et al. 2017), which act together with the AAA-ATPase Vps4 to cut the membrane on one or both sides of the midbody to complete cytokinesis (Adell et al. 2016, Carlton & Baum 2023, Pfitzner et al. 2020, Stuchell-Brereton et al. 2007).

While our understanding of archaeal cell division remains limited, archaea appear to employ a diverse set of division mechanisms (Figure 3). Euryarchaeota and DPANN use an FtsZ-based division mechanism (Baumann & Jackson 1996, Liao et al. 2021a, Margolin et al. 1996, Poplawski et al. 2000, Wang & Lutkenhaus 1996), while many TACK archaea use an ESCRT-III-based division mechanism (Lindås et al. 2008, Samson et al. 2008). In archaea that possess both systems (Ithurbide et al. 2022, Makarova et al. 2010, Pelve et al. 2011), it is not known whether ESCRT and FtsZ work in concert to perform membrane constriction and abscission. However, only ESCRT and ESCRT-associated proteins have been shown to localize to the division plane in these cells (Pelve et al. 2011). Other archaea possess neither system and appear to utilize an as-yet-undiscovered mechanism to divide. For example, members of Thermoproteales do not appear to possess either an FtsZ or an ESCRT machinery (Ettema et al. 2011). They do, however, encode both Crenactin and the associated regulatory Arcadin proteins (Ettema et al. 2011, Izoré et al. 2016). To date, this remains the most likely division machinery in this order. If that holds true, it will be the only archaeal example of a eukaryotic-like actin-based division machinery.

While much of this division machinery may be shared with bacteria and many eukaryotes, the mechanics of archaeal cell division are likely to be profoundly different from those of most bacteria and many eukaryotes (like plants and fungi). As archaea tend to lack a rigid cell wall, the division ring cannot act by guiding cell wall synthesis. Instead, the division ring needs to exert the force required to cut the cell membrane into two. Most archaea possess a flexible cell envelope composed of a protective para-crystalline glycoprotein lattice, termed the surface layer (S-layer), embedded in a single bounding lipid membrane [although exceptions exist, e.g., *Ignicoccus hospitalis*, which has both an outer and an inner membrane (Burghardt et al. 2009)]. These envelope components and their remodeling are discussed in the next section.

### The Nature of the Archaeal Cell Surface

While the protein machinery used to constrict the archaeal cell during division is related to that found in bacteria and eukaryotes, the archaeal membrane is chemically and structurally distinct from that of both bacteria and eukaryotes. With few exceptions, bacterial and eukaryotic membranes are formed from fatty acids linked to *sn*-glycerol-3-phosphate backbones by an ester linkage. In contrast, archaeal lipids are characterized by ether linkages between an *sn*-glycerol-1-phosphate backbone and isoprenoid chains (De Rosa & Gambacorta 1988, Jain et al. 2014b, Koga & Morii 2007). This difference in lipid composition is termed the lipid divide and is proposed to have originated soon after LUCA; however, the exact nature of the membrane of LUCA remains a mystery (Koga et al. 1998, Koonin & Martin 2005, Lombard et al. 2012, Peretó et al. 2004, Sojo et al. 2014). While this divide has often been presented as a gulf too wide to bridge, membranes across the three domains of life share similar polar head groups (Jain et al. 2014a, Koga et al. 1993), which are likely to interact with membrane-associated protein machinery. Moreover, organisms that possess mixed membranes have recently been identified (Villanueva et al. 2021), indicating that different types of lipids can coexist within one membrane. Finally, many archaeal transmembrane proteins appear to have survived the change in lipid composition that accompanied eukaryogenesis without changing their function.

An additional factor that complicates our understanding of archaeal cell division is that the membrane of many archaea is composed of a combination of bilayer-forming diether ~C_20_ lipids and monolayer-forming tetraether ~C_40_ lipids. Tetraether lipids appear to be formed from a linkage of two diether lipids (Lloyd et al. 2022, Zeng et al. 2022), and their presence tends to correlate with low pH and high growth temperature. They are found only rarely in mesophiles like Haloarchaea (Boyd et al. 2013, Tourte et al. 2020). The ratio of diether/tetraether lipids appears to change in response to several environmental factors (Jensen et al. 2015, Lai et al. 2008, Matsuno et al. 2009, Sprott et al. 1991). While the presence of tetraether lipids likely alters the biophysical properties of the membrane, so that more energy is required for remodeling, it is not yet clear whether they form specialized domains in archaeal membranes or how membranes composed of both bilayer and monolayer lipids are remodeled during division.

The S-layer, which coats the lipid membrane of archaea, likely has to be remodeled as cells divide. Although a huge diversity of S-layer structures has been described so far, it is typically composed of up to two protein subunits that self-assemble into two-dimensional arrays of paracrystalline lattices (Bharat et al. 2021, Sleytr et al. 2014). The S-layer is thought to influence cell shape, cell size (Pum et al. 1991, Zhang et al. 2019), and cell-cell contacts (Burghardt et al. 2009) as well as to protect cells from viral infection and the environment (Zink et al. 2019). The S-layer also generates a pseudo-periplasmic space that is a known site for cellular biochemical reactions (Albers & Meyer 2011). Because the S-layer performs an important set of functions, it is unclear how it can be remodeled during division without its integrity being compromised. Cell division poses a challenge in S-layer remodeling as surface curvature changes drastically during membrane constriction. Studies looking at S-layers in several model archaea, including *H. volcanii* and *S. acidocaldarius*, have demonstrated that the S-layer can tolerate large changes in shape. In one study, *H. volcanii* S-layer lattices curved through the incorporation of pentamers into the normally hexagonal lattice (von Kügelgen et al. 2021). Importantly, this process can occur without new insertion, enabling S-layer remodeling during membrane deformation. The fact that the *S. acidocaldarius* S-layer structure utilizes two S-layer proteins, a membrane anchor (SlaB) and a lattice-forming canopy (SlaA), may also provide the flexibility required for large-scale membrane remodeling (Gambelli et al. 2019, 2024).

Interestingly, there is also evidence that the S-layer may aid division in some cells. *S. islandicus* and *S. solfataricus* cells lacking an S-layer exhibit cell division defects (Zhang et al. 2019, Zink et al. 2019). However, the molecular and biophysical roles of the S-layer in cell division have yet to be established. Moreover, in *H. volcanii*, it appears that new S-layer components are inserted at the site of division (Abdul-Halim et al. 2020), which may accommodate the increase in surface area required when a cell divides into two (~2^1/3^ times for spherical cells with constant total volume). Finally, glycosylation of the S-layer proteins has been implicated in cell division, as inhibition of glycosylation with the drug tunicamycin results in cell division defects in Sulfolobales (Hjort & Bernander 2001, Palmieri et al. 2013, Peyfoon et al. 2010).

### The Role of the Cytoskeleton in Archaeal Cell Division

The forces required for division are mostly generated via nucleotide hydrolysis and the binding of protein polymers to the membrane. Several reviews have recently explored the intricacies of the archaeal FtsZ and ESCRT systems (Blanch Jover & Dekker 2023, Carlton & Baum 2023, Ithurbide et al. 2022). Here, we compare and highlight key similarities and differences between the ways in which bacteria, eukaryotes, and archaea divide.

#### FtsZ-driven cell division

FtsZ, a homolog of eukaryotic tubulin, is the most conserved cell division protein in bacteria (Margolin 2005). FtsZ polymerizes in a GTP-dependent manner to form a dynamic ring at the site of cell division that acts as a scaffold to recruit a host of other cell division factors, including the divisome complex. Membrane deformation in division is driven by local remodeling and de novo synthesis of the peptidoglycan cell wall at the site of the FtsZ ring. Key players in the assembly of the bacterial divisome are FtsZ, its membrane tethers ZipA and SepF (Hale & de Boer 1997, Liu et al. 1999), and FtsA—an actin homolog that regulates divisome assembly and activation (Cameron & Margolin 2024). While either ZipA or FtsA suffices for FtsZ ring formation, both are required for the recruitment of downstream proteins (Chen & Beckwith 2001, Hale & de Boer 2002). Essential downstream proteins include a DNA linker/translocase (FtsK); peptidoglycan glycosyltransferases (FtsW/I); FtsEX, which regulates cell wall hydrolysis; and further scaffolding proteins such as FtsQ/L/B and FtsN, which appear to act as the trigger for cell wall synthesis and membrane constriction. In-depth reviews on bacterial cell division are available (Mahone & Goley 2020, McQuillen & Xiao 2020).

By comparison, archaeal FtsZ systems are less well characterized and are further complicated by the fact that most archaea do not possess a peptidoglycan-like cell wall. Importantly, in those that do, such as *Methanobrevibacter smithii*, a single FtsZ homolog (FtsZ1) (Steenbakkers et al. 2006, Visweswaran et al. 2011) is thought to coordinate pseudopeptidoglycan cell wall synthesis with cell division in a similar manner to the bacterial divisome. In this case, the cell wall synthesis machinery appears to have been acquired from bacteria via horizontal gene transfer (Ithurbide et al. 2022, Subedi et al. 2021). By contrast, most archaea lack a cell wall and encode for two FtsZ homologs, FtsZ1 and FtsZ2. These homologs appear to have different functions in division, with FtsZ1 acting in a scaffolding/template role, while FtsZ2 appears to be involved in membrane constriction (Liao et al. 2021a). This finding suggests that division in a soft cell requires the assembly of the ring prior to force generation. The best-studied example of the archaeal dual FtsZ system is *H. volcanii*. In *H. volcanii*, FtsZ1 forms filaments in a GTP-dependent manner at the site of cell division (Liao et al. 2021a). FtsZ2 is then recruited to the FtsZ1 ring and anchored to the membrane by SepF, which appears to be ancestral and is found in all archaea that encode FtsZ (Pende et al. 2021). Both FtsZ2 GTPase activity and the presence of SepF are essential for the remodeling and constriction of the FtsZ1/FtsZ2 ring (Figure 3). How force is generated by the dual FtsZ system remains an open question. Interestingly, in addition to a scaffolding role, FtsZ1 also appears to have a role in cell shape and cell envelope growth (Liao et al. 2021a).

Many archaea contain additional FtsZ/tubulin homologs (Duggin et al. 2015, Vaughan et al. 2004) that fall into different classes. Haloarchaea, which often display pleomorphic cell shape phenotypes (including flattened rectangular prisms or triangles), frequently contain tubulin homologs (Burns et al. 2004, Takashina et al. 1990, Walsby 1980). One of these, CetZ, is unique to archaea (Duggin et al. 2015). CetZ forms dynamic filaments in *H. volcanii* that localize to both the mid-cell and regions of high membrane curvature. It appears to have a primary role in the regulation of cell shape rather than cell division (Duggin et al. 2015). A number of questions can be raised about the functional association of the shape-regulating machinery (CetZ) and the envelope growth machinery (Sec/ArtA/PssA/PssD) with the FtsZ1/FtsZ2 division rings, as they all localize at mid-cell in *H. volcanii* (Abdul-Halim et al. 2020, Duggin et al. 2015). In particular, FtsZ1 has been proposed to function as a coordinator of cell shape determination and division. It is intriguing to consider whether S-layer biogenesis is functionally coupled to FtsZ-mediated constriction in archaea in a way that mirrors the coupling of cell wall growth to FtsZ constriction in bacteria.

There are multiple open questions regarding the function and composition of archaeal FtsZ systems. First, FtsZ does not seem to be essential for cell survival, as both FtsZ1 and FtsZ2 can be deleted in *H. volcanii*. Despite suffering severe division phenotypes, these cells remain viable (Liao et al. 2021a). In contrast, SepF appears to be essential in *H. volcanii* (Nußbaum et al. 2021). Second, it is not clear how the site of FtsZ-mediated division is determined in archaea. In bacteria, the positioning of the FtsZ ring is well characterized. In *E. coli*, for example, cell polarity and the positioning of the FtsZ ring are established by an oscillating Turing-type patterning of the MinD/E system (Rowlett & Margolin 2015, Walsh et al. 2019, Yu & Margolin 1999), although other Z-ring localization systems may also exist (Bailey et al. 2014, Rodrigues & Harry 2012). In archaea, however, homologs of the Min system either are absent or do not appear to contribute to cell division or polarity establishment (Ithurbide et al. 2022). Instead, MinD homolog oscillations in *H. volcanii* appear to determine the positioning of the archaellum for cell motility (Nußbaum et al. 2020). Despite the lack of a Min system, Walsh et al. (2019) have shown that division plane localization in *H. volcanii* is consistent with a Turing-type pattern as it can be predicted from cell shape and is not dictated by the orientation of the previous division plane.

#### ESCRT-driven cell division

While the ESCRT system mediates the final stages of membrane constriction and abscission in eukaryotes, archaeal homologs of ESCRT-III mediate the entire process of constriction and abscission in archaea (Carlton & Baum 2023). ESCRT-III proteins form homo- or heteropolymers that co-assemble on the membrane. Recent data suggest that these ESCRT-III polymers assemble in a stepwise manner, as one triggers the assembly of the next before individual polymers are disassembled by Vps4 (Hurtig et al. 2023, Jiang et al. 2022, Meadowcroft et al. 2022, Pfitzner et al. 2020). The sequential polymerization and turnover of composite ESCRT-III filaments by Vps4 remodel membrane necks to induce the final stages of membrane constriction and abscission (Adell et al. 2016, Pfitzner et al. 2020, Stuchell-Brereton et al. 2007).

The best-characterized archaeal ESCRT-III system is the Sulfolobales Cdv (cell division) system (Lindås et al. 2008, Samson et al. 2008). In *S. acidocaldarius*, cell division is driven by three ESCRT-III homologs (CdvB, CdvB1, and CdvB2) working in concert with the AAA ATPase Vps4 and a TACK-specific protein, CdvA (Figure 3). Like archaeal FtsZ1 and FtsZ2, the Cdv system consists of an initial scaffold/template ring (CdvA and CdvB), which acts to recruit the constricting rings (CdvB1 and CdvB2) (Lindås et al. 2008, Samson et al. 2008). It is thought that the membrane-binding CdvA first defines the plane of division on which the non-membrane-binding CdvB can polymerize (Samson et al. 2011). The CdvB ring then serves as a template to recruit both CdvB1 and CdvB2 [which can directly interact with the cell membrane (Blanch Jover et al. 2022)], forming distinct, spatially organized filaments at the plane of division (Hurtig et al. 2023). Once the CdvB1 and CdvB2 rings have assembled, CdvB is removed from the composite ring by Vps4 and degraded in a proteasome-dependent manner (Tarrason Risa et al. 2020). The selective removal of CdvB from composite division rings is likely due to the relatively high affinity of Vps4 for CdvB (Samson et al. 2008). CdvB removal and degradation appear to be essential for cell constriction, as proteasome inhibition locks the ring in its preconstriction, CdvB-positive state (Hurtig et al. 2023, Tarrason Risa et al. 2020). However, the molecular cue for CdvB ring disassembly remains unknown.

After degradation of the noncontractile CdvB ring, the CdvB1 and CdvB2 rings constrict the membrane in a Vps4 activity–dependent manner (Blanch Jover et al. 2022, Hurtig et al. 2023, Tarrason Risa et al. 2020). The energy for membrane remodeling is likely provided by a combination of Vps4-dependent filament remodeling as well as elastic energy stored by CdvB1 and CdvB2 polymers, which are thought to have a higher preferred intrinsic curvature than CdvB (Hurtig et al. 2023). While both CdvB1 and CdvB2 appear to be essential for *S. islandicus* (Liu et al. 2017, Zhang et al. 2018), *S. acidocaldarius* cells lacking either CdvB1 or CdvB2 can still divide but with higher failure rates and abnormal morphology. The deletion of CdvB1 has a mild division defect, with occasional failure during constriction and abscission. Cells lacking CdvB2, on the other hand, frequently display division asymmetries, potentially due to the slippage of the constricting ring away from the cell center (Pulschen et al. 2020). Superresolution imaging of constricting *S. acidocaldarius* cells has shown that CdvB2 rings concentrate at the center of the membrane neck with the highest membrane curvature flanked by a wider zone of CdvB1 polymers (Hurtig et al. 2023). This distinct spatial pattern suggests that the CdvB2 polymer prefers a highly curved membrane, while CdvB1 polymers can bind to membranes with a larger range of curvatures. When acting together, the differential localizations and curvature preferences of CdvB1 and CdvB2 may contribute to the high fidelity of cytokinesis by facilitating the large change in membrane curvature (from a radius of ~1 µm to <100 nm) and by preventing the misplacement of the division plane during constriction and abscission. While the individual roles of each Cdv protein have recently started to emerge, several key questions remain. These include the fate of CdvA during division, how the division plane is initially determined, and how this process of cell division is coupled spatially and temporally with DNA segregation and S-layer remodeling.

#### Asgard cell division

Asgard archaea, the closest relatives of eukaryotes, have only recently been isolated and have not yet been imaged during division. It will be exciting to see how they divide. While little is known for certain, some clues can be gained from their genome. Importantly, in nearly all cases, Asgard archaeal cells possess two ESCRT-III homologs, together with Vps4, but lack CdvA homologs that are essential for ESCRT-III-mediated cell division in Sulfolobales. In addition, the ESCRT-III genes are present in loci alongside homologs of ubiquitin and ESCRT-I and -II proteins (Hatano et al. 2022), implying that these proteins play roles analogous to the eukaryotic ESCRT system in transmembrane protein proteostasis. Whether Asgard ESCRT-III homologs are involved in the last step of cytokinesis to abscise membranes, like their eukaryotic counterparts, remains to be tested. However, they possess one or two copies of FtsZ along with SepF (Pende et al. 2021), implying that Asgard archaea use this machinery for division. Some Asgard archaea also possess actin and tubulin homologs, which may have to be disassembled at division or which may aid this process. The only two Asgard archaeal species cultivated to date also appear to lack a complete S-layer (Imachi et al. 2020, Rodrigues-Oliveira et al. 2023), implying that its role in cell shape control and division may be less important than it is in other archaea.

## Conclusion

Studies over the past two decades have provided a glimpse of how archaea replicate their genome and divide. Features that are shared across different archaeal species help reveal the universal physical and molecular principles that govern this fundamental cellular process. At the same time, system-specific differences provide insights into the way cells can achieve the same goal via different means and highlight the diversity of biophysical challenges archaeal cells face when dividing.

Recent experimental and phylogenetic studies have started to reveal the rich cell biology of archaea. However, our understanding remains poor. This is due in part to the technical challenges faced when working with organisms that require extreme culturing conditions, limiting the use of live-cell imaging, and the availability of genetic tools, fluorescent probes, and tags. Moreover, while many archaea have been identified by environmental sampling and metagenomics (e.g., most DPANN and Asgard archaea), only a few have been successfully cultured in a lab environment. As these challenges are overcome through the development of novel tools and culturing methods, it will be possible to address the many outstanding questions in archaeal biology, giving us fresh insights into the origins of cell cycle control in eukaryotes.
